# Seasonal Distribution of Atmospheric Coarse and Fine Particulate Matter in a Medium-Sized City of Northern China

**DOI:** 10.3390/toxics10050216

**Published:** 2022-04-25

**Authors:** Xin Zhang, Bianhong Zhou, Zhiyu Li, Yue Lin, Lijuan Li, Yuemei Han

**Affiliations:** 1 Key Laboratory of Aerosol Chemistry and Physics, State Key Laboratory of Loess and Quaternary Geology, Institute of Earth Environment, Chinese Academy of Sciences, Xi’an 710061, China; zhangxin@ieecas.cn (X.Z.); lizhiyu@ieecas.cn (Z.L.); linyue@ieecas.cn (Y.L.); lilijuan@ieecas.cn (L.L.); 2 Chinese Academy of Sciences Center for Excellence in Quaternary Science and Global Change, Xi’an 710061, China; 3 School of Human Settlements and Civil Engineering, Xi’an Jiaotong University, Xi’an 712000, China; 4 Shaanxi Key Laboratory of Disaster Monitoring and Mechanism Simulation, Department of Geography and Environmental Engineering, Baoji University of Arts and Sciences, Baoji 721013, China; bhz620@163.com

**Keywords:** aerosol particles, size distribution, seasonal variation, meteorology influence, source

## Abstract

Atmospheric particulate matter (PM) was measured continuously at an urban site in Baoji city in northern China in 2018 to investigate the seasonal distribution characteristics. Coarse PM (PM_2.5–10_) was more prevalent in spring, substantially due to the regional transport of dust. High loadings of coarse PM were found at night compared to daytime, which could result from high production and unfavorable dispersion conditions. Fine PM (PM_2.5_) constituted, on average, 54% of the total PM mass concentration, whereas it contributed more than 97% of the total PM number concentration. The number and mass concentrations of fine PM increased substantially in the winter, which was possibly due to the enhanced production of atmospheric secondary processes and coal combustion. Precursor gaseous pollutants and meteorology greatly influenced the PM distributions. Fine PM was associated more strongly with gas pollutants than coarse PM, which suggested that it largely originated from secondary production and combustion sources. High relative humidity appeared to promote the production of fine PM, whereas it facilitated the removal of coarse PM. This study highlights that different air-pollution control strategies should be used for coarse and fine PM according to the distribution characteristics and influencing factors in similar medium-sized urban areas.

## 1. Introduction

Atmospheric particulate matter (PM) is the dominant air pollutant in many cities in China with rapid economic development and expanded urbanization [1,2]. High loadings of atmospheric PM can lead to substantial degradation of air quality, reduced visibility, and adverse impacts on human health [3,4]. They also significantly contributed to heavy haze pollution episodes that frequently occurred, in particular during winter, in the past two decades [5,6,7]. Atmospheric PM of different sizes usually has distinct characteristics in terms of its sources, production and removal processes, and environmental impacts. The mass distributions of atmospheric PM are mostly dominated by two modes: the fine accumulation mode and coarse mode [8]. Fine PM is largely generated from primary combustion emissions and secondary production from gas to particle conversion and condensation, whereas coarse PM is mostly produced by mechanical processes. Understanding the distribution characteristics of atmospheric PM would provide valuable information on its sources and production processes, which is essential to developing effective air pollution control strategies.

Baoji is a medium-sized city in northern China with a population of 3.76 million in 2019 (http://tjj.shaanxi.gov.cn/upload/n2020/indexch.htm, accessed on 4 March 2021). It is among the 11 major cities of the Fenwei basin, which has been designated as a key region for air pollution control in China. Baoji is situated at the west end of the Fenwei basin. Long-lasting and widespread haze pollution episodes have frequently occurred in this region in recent years [9]. The basin terrain with substantial emissions is particularly unfavorable for the dispersion of air pollutants. Local governments have taken great efforts to improve the air quality in past years, such as shutting down or migrating industrial factories and restricting traffic. The air pollution situation, however, remains poor, partly due to the lack of comprehensive knowledge and profound understanding of the complex contributing factors. The chemical composition and source apportionment of atmospheric PM have been investigated in Baoji city by some prior studies thus far [10,11,12,13], but most of those studies were short-term studies and rarely focused on mass distributions of PM. A year-round study on the distribution and sources of PM pollutants is important for a comprehensive understanding of the contributing factors and helpful for more targeted air pollution control.

This study aims to understand the seasonal distribution characteristics of atmospheric coarse and fine PM in Baoji city and to identify the key contributing factors. The size-resolved number and mass concentrations of atmospheric PM were continuously monitored at an urban site in Baoji city from February to December 2018. The temporal variation trends of coarse and fine PM were characterized across the year. The potential contribution sources associated with coarse and fine PM were also discussed. The comprehensive influences of precursor gaseous pollutants and meteorological factors on the mass concentration and seasonal distributions of PM were further examined.

## 2. Experimental Methods

Atmospheric PM was monitored continuously on the rooftop of a six-floor office building (107.20° E, 34.35° N; approximately 20 m above ground level) on the main campus of Baoji University of Arts and Sciences, Baoji, Shaanxi, China. The measurement duration was from 10 February to 30 December 2018. The sampling site was situated in an urban area mostly surrounded by the university campus and residential blocks. Several arterial roads were located in the south and west of the sampling site and usually with medium traffic volumes. There were no apparent industrial plants or other major pollution sources nearby.

The size-resolved number and mass concentrations of atmospheric PM from 0.25 up to 32 µm in aerodynamic diameter were measured using an optical environmental particle monitor (EDM-180, GRIMM Aerosol Technik, Germany) [14]. The measuring principle was as follows: ambient air was sampled through the inlet head at a flow rate of 1.2 L min^−1^ without particle-size-selective segregation; the sampled air was then led directly to the measuring cell, where a laser diode served as a scattering light source; PM number was counted according to the scattering light pulse, while PM size was classified by the intensity of scattering light signal. The number concentrations of PM at 31 different size channels were obtained from the measurement. The monitoring data were acquired at 5 min intervals. Surface area and volume concentrations were calculated, respectively, by π*d_p_*^2^*N* and π*d_p_*^3^*N*/6, in which *d_p_* is the aerodynamic diameter and *N* is the PM number concentration of each size bin. PM mass concentrations were calculated from the number–size distributions using the density factors established for urban environments by assuming spherical particles [14]. The detection range of PM mass concentration was from 0.1 to 6000 μg m^−3^.

Hourly data of meteorological factors, including temperature, relative humidity (RH), wind speed and direction, precipitation, and horizontal visibility, were obtained from the Meteorology Bureau of Baoji city for the entire monitoring period. The meteorology station (107.14° E, 34.36° N) is located approximately 2.5 km southwest of the sampling site. The hourly mass concentrations of PM and precursor gaseous pollutants, including PM_2.5_, PM_10_, O_3_, SO_2_, NO_2_, and CO, were obtained from the China National Environmental Monitoring Centre (http://www.cnemc.cn, accessed on 1 January 2021). The gaseous pollutants were measured at the nearest national air quality monitoring station that was on the same building rooftop and right beside the sampling site. Moreover, the daily backward trajectories of air masses arrived at the sampling site (height: 500 m above ground level; duration: 72 h; start time: 9:00 a.m. each day) were calculated over the studied period using the Hybrid Single Particle Lagrangian Integrated Trajectory model [15,16].

## 3. Results and Discussion

### 3.1. Seasonal Variation Profiles of PM

**Figure 1:**
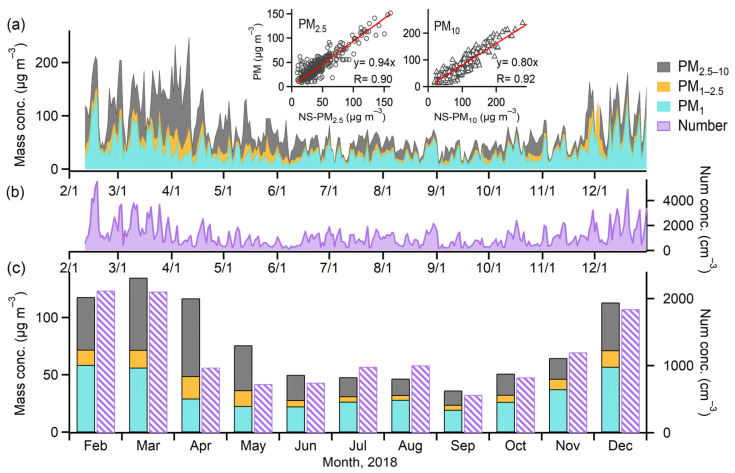
Temporal variations of the (**a**) mass and (**b**) number concentrations of PM at daily resolution and (**c**) the monthly mean PM concentrations over the studied period. The two small panels in (**a**) present the correlations of PM_2.5_ and PM_10_ mass concentrations measured by the EDM-180 particle monitor and those obtained from the national air quality monitoring station.

The daily and monthly variations of PM mass and number concentrations over the studied period are presented in Figure 1. The mass concentrations were, on average, 77 ± 61 μg m^−3^ (mean ± 1σ standard deviation) for PM_10_, 45 ± 30 μg m^−3^ for PM_2.5_, and 35 ± 26 μg m^−3^ for PM_1_ across the year. Here, PM_10_, PM_2.5_, and PM_1_ correspond to the PM with aerodynamic diameters less than 10, 2.5, and 1 µm, respectively. These values are much higher compared to the annual air quality limit standards for PM_2.5_ and PM_10_ (5 and 15 μg m^−3^, respectively) published by the World Health Organization (2021). In general, the PM mass concentrations were substantially higher in the spring and winter seasons compared to those in the summer and fall seasons. The total mass concentrations were significantly composed by those of coarse particles PM_2.5–10_ during springtime, whereas it was dominated by fine particles of PM_2.5_ during wintertime. The mass concentrations of PM_2.5_ and PM_10_ measured by the EDM-180 particle monitor in this study correlated strongly overall with those from the national air quality monitoring station (*r* of 0.90 and 0.92, respectively) (the small panels in Figure 1a). Nevertheless, the former were slightly lower compared to the latter. This was possibly due to the uncertainties associated with the different principles of monitoring devices; that is, a tapered element oscillating micro-balance was used at the national air quality monitoring station. 

The total number concentration of PM from 0.25 to 10 µm was, on average, 1172 ± 1018 cm^−3^ for the studied period. Higher PM number concentrations were mostly observed in winter and early spring (e.g., December, February, and March) compared to those of other periods (April to November) (Figure 1b,c). Fine PM contributed, on average, more than 97% of the total PM number concentration throughout the year. This characteristic is different from the large contributions of mass concentration from both coarse and fine PM. The number concentration reported herein would be even higher if accounting for PM below 0.25 µm, which was not available due to the detection limit of the optical particle monitor.

**Figure 2:**
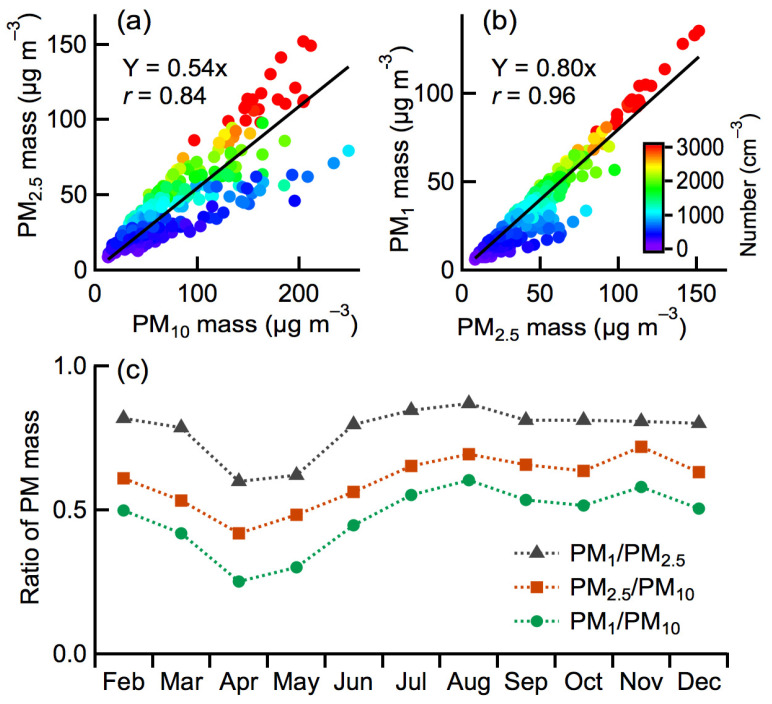
Correlations of the mass concentration of (**a**) PM_2.5_ vs. PM_10_ and (**b**) PM_1_ vs. PM_2.5_ over the studied period, and (**c**) the monthly averages of the mass concentration ratios of PM_1_/PM_2.5_, PM_2.5_/PM_10_, and PM_1_/PM_10_.

Figure 2 presents the correlations and ratios among the mass concentrations of PM_10_, PM_2.5_, and PM_1_ over the studied period. The mass concentrations of PM_2.5_ correlated highly overall to those of PM_10_, with a correlation coefficient *r* of 0.84 (Figure 2a). PM_2.5_ constituted, on average, 54% of PM_10_ in the mass concentration. In contrast, the mass concentrations of PM_1_ correlated more strongly to those of PM_2.5_ (*r* of 0.96; Figure 2b). PM_1_ constituted, on average, 80% of PM_2.5_ in the mass concentration. Interestingly, with the increase in the PM number concentration, the mass fractions of PM_2.5_ to PM_10_ and PM_1_ to PM_2.5_ were both increased, as indicated by the colored dots in Figure 2a,b. This is consistent with the large population of fine PM. Moreover, the mass concentration ratios of PM_1_/PM_2.5_, PM_2.5_/PM_10_, and PM_1_/PM_10_ varied in different seasons (Figure 2c). The ratios all substantially decreased during the springtime of April and May compared to those in other periods. On the other hand, slightly higher ratios of PM_1_/PM_2.5_ were found during the summertime of July and August, whereas higher ratios of PM_2.5_/PM_10_ and PM_1_/PM_10_ were found in both August and November. The higher fractions of fine PM in total PM_10_ mostly resulted from the lower mass concentrations of coarse PM (Figure 1c) and possibly the enhanced contributions of fine PM from secondary production.

**Figure 3:**
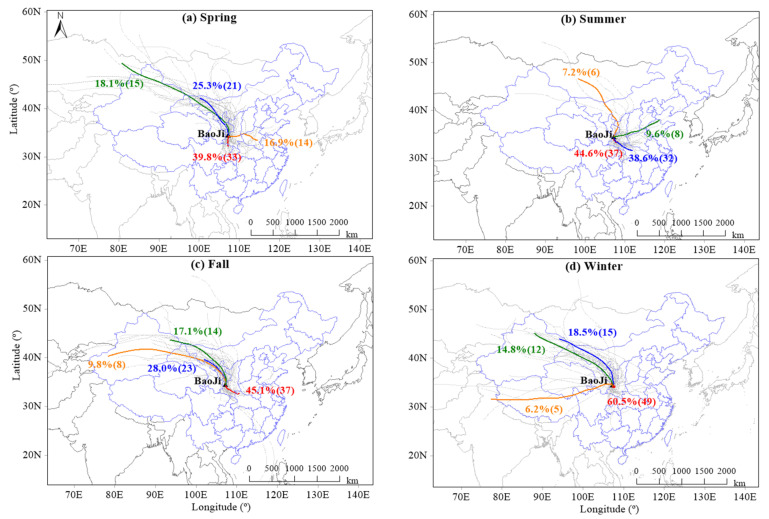
The daily backward trajectories of air masses and the cluster analysis in the four seasons of spring (March–May), summer (June–August), fall (September–November), and winter (December–February) of 2018 in Baoji city. The duration of each trajectory was 72 h. The solid colored lines represent air masses with different origins based on the cluster analysis. The values represent the percentage and the number of air mass trajectories with different origins.

The results discussed above represent the typical characteristics of PM distributions in Baoji city in different seasons across the year. This area was frequently polluted by coarse PM in spring, which was mostly due to the long-range transport of dust from desert regions in northwest China [17,18]. Figure 3 presents the daily backward trajectories of air masses and the cluster analysis in the four seasons of Baoji city in 2018. A great portion of air masses originated from the northwest region in spring, which accounted for approximately 43.4% of the total amount (Figure 3a) and was much higher than those of other seasons (7.2–33.3%). Air masses from the surrounding areas of the south and southeast regions also greatly contributed (39.8% and 14%). Moreover, the wind speed was relatively higher in the spring compared to other seasons (presented later in Section 3.3), which would also facilitate the agitation and resuspension of coarse PM from local sources such as construction and road dust. In contrast, both the coarse and fine PM, in general, presented relatively low concentrations in summer and fall. This is possibly, at least to a large extent, due to the absence of substantial emission and the production of PM and its precursors from coal combustion, together with the favorable dispersion conditions for the removal of air pollutants. Air masses were also mostly from the southeast regions in the summer and fall (Figure 3b,c).

Baoji is a medium-sized city in northern China, where coal combustion is commonly utilized for heating in the winter. The heating period is usually from mid-November to mid-March of the next year. Primary emissions of coal combustion contribute approximately 10% of submicron organic aerosols in Baoji city during the heating season [10], in addition to the contributions of coal combustion to coarse PM and atmospheric secondary production of fine PM. Other pollution sources, such as heating-related biomass burning from the surrounding rural area, would also contribute to the increased PM concentration in winter [19]. A great number of air pollutants might be contributed by sources from the local and surrounding areas in winter, as indicated by the air mass origins (60.5% in Figure 3d). Given the distinctive characteristics of PM distributions, further PM pollution control strategies should target the specific contributing factors in different seasons.

### 3.2. Size Distributions of PM

**Figure 4:**
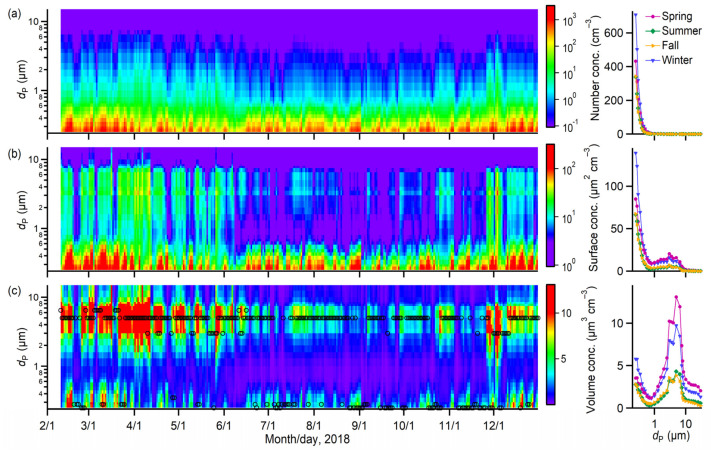
Size-resolved (**a**) number, (**b**) surface area, and (**c**) volume concentrations of PM over the studied period and their seasonally averaged size distributions. Results presented in the image plots were averaged for daily data. The black circles in (**c**) represent the mode diameters of PM volume concentrations.

Figure 4 presents the size-resolved number, surface area, and volume concentrations of PM over the studied period together with the seasonally averaged size distributions. The daily mean of total PM_10_ number concentration was in the range of 138 to 5540 cm^−3^. The PM number concentration was primarily dominated by fine PM throughout the year, accounting for 97–99% of the total PM_10_ amount. The number concentration of PM at a diameter below 0.4 µm was particularly abundant compared to larger sizes. This result is consistent with prior studies at various locations wherein small particles usually have much larger populations [20,21]. The highest PM number concentration was found in winter, which might be significantly contributed by the enhanced production of secondary pollutants from coal combustion for heating. Moreover, the PM surface area concentration was significantly contributed by fine PM (on average 87%) and mostly due to their large populations. The contribution of coarse PM (on average 13%) to the total surface area concentration increased slightly because of its large size, compared to the small contribution to the total number concentration (i.e., less than 3%). In contrast, the PM volume concentrations were mostly contributed by coarse PM, especially those with mode diameters of approximately 5 µm (see the black circles in Figure 4c). Coarse PM constituted, on average, 54% of total PM_10_ volume concentration, compared to 46% for fine PM. The PM volume concentrations were comparable between summer and fall, and the averaged size distribution trends were nearly similar to those in winter and spring despite their relatively low concentrations.

**Figure 5:**
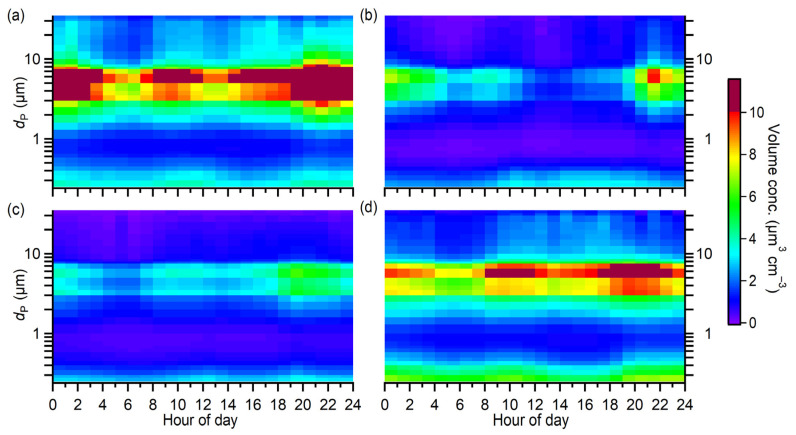
Diurnal profiles of the size-resolved PM volume concentrations for individual seasons: (**a**) spring, (**b**) summer, (**c**) fall, and (**d**) winter.

The diurnal profiles of size-resolved PM volume concentrations were further examined for individual seasons, as presented in Figure 5. Coarse PM, on average, had the highest volume concentration in spring, whereas fine PM had the highest volume concentration in winter. For coarse PM across the four seasons, they were most abundant during the nighttime, that is, from approximately 18:00 to 05:00 of the next day in local time (LT). There was also a small peak during the daytime from around 08:00 to 12:00 LT. On the contrary, fine PM was abundant at noon and night. Photochemical reactions of precursor gaseous air pollutants could substantially contribute to the production of fine PM during the daytime. The continuous growth of fine PM by coagulation, condensation, and further oxidation processes, together with the unfavorable disperse conditions (as discussed in Section 3.3), might greatly contribute to the production of coarse PM during the nighttime. The abundance of coarse and fine PM might also be primarily and secondarily contributed by traffic vehicle emissions, particularly during the morning and evening rush hours.

### 3.3. Influences on PM Concentration and Distribution

**Figure 6:**
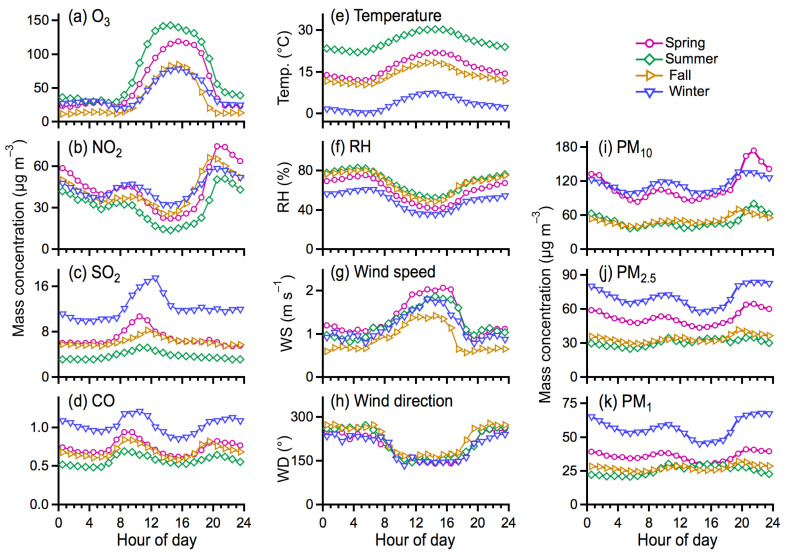
Diurnal profiles of (**a**–**d**) the precursor gaseous pollutants of O_3_, NO_2_, SO_2_, and CO and (**e**–**h**) the meteorological parameters of temperature, relative humidity, wind speed, and wind direction, and (**i**–**k**) the mass concentrations of PM_10_, PM_2.5_, and PM_1_ for spring, summer, fall, and winter seasons.

The origins of air pollutants and meteorological conditions appear to be the key factors governing the concentration and distribution of PM. Figure 6 presents the diurnal profiles of gaseous precursor pollutants, meteorological parameters, and PM mass concentrations for individual seasons. Both the precursor gases and meteorological parameters turned out to have nearly similar diurnal variation trends among the four seasons (Figure 6a–h), despite their different levels, which are, to a certain extent, consistent with the similar variation trends of PM mass concentrations (Figure 6i–k). This could have resulted from the relatively similar contributions from pollution sources and meteorological conditions in the studied region across the year. O_3_ concentration increased substantially during the daytime in summer (Figure 6a), which, nevertheless, usually corresponded to low PM concentrations. Strong solar radiation in summer could prompt the enhanced O_3_ production from photochemical reactions of volatile organic compounds and NO_x_ [22,23]. The low PM concentration also could be closely associated with the higher boundary layer height during summertime.

NO_2_ concentration showed two prominent peaks in the morning (07:00–09:00 LT) and nighttime (19:00–21:00 LT), respectively. This is consistent with the large emissions of NO_x_ from vehicle exhaust during traffic rush hours. High RH might promote the aqueous phase reaction of NO_x_ for the secondary formation of nitrates [24], which could partly contribute to the high PM concentration, particularly at nighttime (Figure 5). Extra-high concentrations of SO_2_ and CO were exclusively found in winter compared to other seasons, which is most likely attributed to the substantial contributions of air pollutants from coal combustion for heating [25]. CO might also be contributed partly by vehicle emissions, which is indicated by the similar peaks of those of NO_2_ at nighttime.

**Figure 7:**
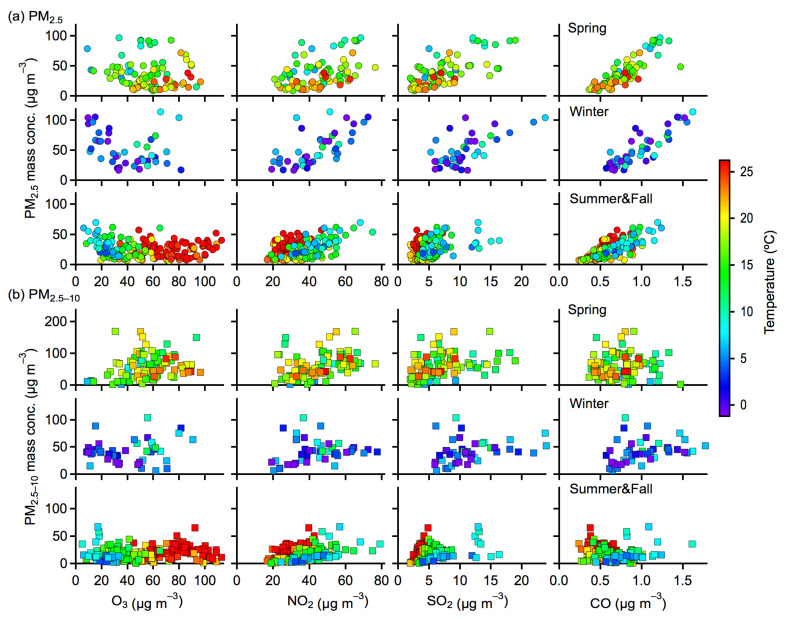
Correlations of the mass concentrations of (**a**) PM_2.5_ and (**b**) PM_2.5–10_ versus gaseous pollutants (O_3_, NO_2_, SO_2_, and CO). The markers were colored by the air temperature. Results presented here were averaged for daily data.

The comprehensive influences of precursor gases and meteorological conditions on the PM distributions were examined to gain further insights. Figure 7 presents the correlations of PM mass concentrations as functions of precursor gases for different seasons. The results in summer and fall were discussed together here because of their similar PM levels and variation trends. For fine PM, the mass concentrations generally exhibited positive increasing trends with increasing NO_2_, SO_2_, and CO concentrations, especially in spring and winter (Figure 7a). This most likely resulted from the enhanced secondary production of fine PM in the presence of high concentrations of precursor gases. On the other hand, fine PM concentrations exhibited slightly decreasing trends with an increasing O_3_ concentration. This can be explained by the fact that more O_3_ could be produced due to the reduced scavenging of hydroperoxyl and NO_x_ radicals at low PM concentrations [26]. Another possible cause is that low PM concentration would result in less scattered radiation and thus increase the photolysis frequencies of nitrogen dioxide for producing O_3_ [27]. For coarse PM, however, the mass concentrations were more broadly distributed with those of precursor gases (Figure 7b). There were no clear correlation trends found with increasing precursor gases concentrations compared to those of fine PM. Therefore, the influences of precursor gases on PM concentrations greatly depended on the particle sizes, which could mostly be associated with the different formation and production processes of the coarse and fine PM.

**Figure 8:**
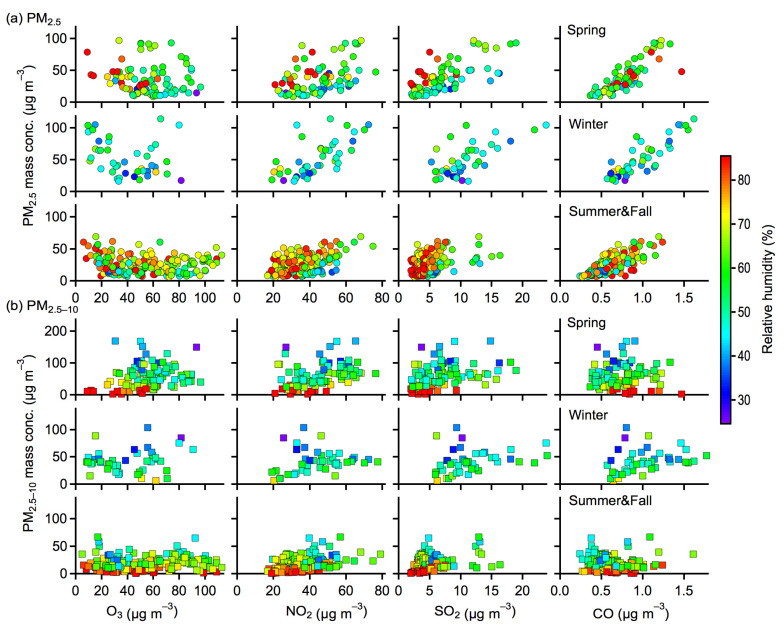
Correlations of the mass concentrations of (**a**) PM_2.5_ and (**b**) PM_2.5–10_ versus gaseous pollutants (O_3_, NO_2_, SO_2_, and CO). The markers were colored by the relative humidity in the air. Results presented here were averaged for daily data.

The meteorological factors of temperature and RH also largely influenced the PM distributions, as indicated by the colored markers in Figure 7 and Figure 8. For PM versus NO_2_, SO_2_, and CO, higher temperatures usually corresponded to higher concentrations of coarse and fine PM in summer and fall, whereas such trends were not clear in spring and winter (see Figure 7). It was likely that photochemical reactions were substantially enhanced at higher temperatures for the production of PM in summer and fall, despite the high temperature being able to facilitate the partitioning of semivolatile compounds into the gas phase and possibly reduce the amount of PM. In the cold seasons of winter and spring, however, temperature inversion occurred frequently, which could hinder the atmospheric vertical convection to a certain extent and thus was favorable for the accumulation and production of air pollutants. A lower temperature could also promote the partitioning of semivolatile compounds into the particle phase and result in an increase of PM concentrations in cold seasons. Higher RH values usually corresponded to higher concentrations of fine PM for different seasons, in particular for PM associated with NO_2_ and SO_2_ (Figure 8a). This result could be explained by the enhanced secondary production of fine PM and the hygroscopic growth at higher RH [28,29]. In contrast, relatively low concentrations of coarse PM were found at higher RH values for different seasons (Figure 8b). It is most likely that the depositional removal or washing out of coarse PM was substantially enhanced at higher RH levels.

## 4. Summary and Implications

The distribution characteristics of atmospheric coarse and fine PM were investigated at an urban site in Baoji city in northern China in 2018. Coarse PM was most abundant in spring and winter and was also relatively more abundant at nighttime than in the daytime across the year. Fine PM constituted a substantial portion of the total PM mass concentration, 54% on average, whereas it constituted more than 97% of the total PM number concentration over the study period. The number and mass concentrations of fine PM greatly increased in wintertime compared to other seasons, which is most likely associated with the primary and secondary productions of air pollutants from coal combustion for heating. Both the coarse and fine PM had relatively low mass and number concentrations in summer and fall. The PM distributions were greatly influenced by the precursor gaseous pollutants and meteorological factors. Fine PM were more correlated to the gas pollutants of NO_2_, SO_2_, and CO than coarse PM, possibly due to large contributions from atmospheric secondary processes. The production of fine PM appeared to be promoted at high relative humidity and low temperature, whereas coarse PM was more likely removed at high relative humidity. This study highlights that different pollution-control strategies should be taken for coarse and fine PM according to their distribution characteristics of individual seasons in Baoji city and the surrounding area. The current work is a typical case study of the distribution characteristics of PM in Baoji city, which provides a reference for other medium-sized cities in the Fenwei basin and other regions. Future studies should also investigate the compositional (especially organic components at molecular levels) and other physical and chemical properties of coarse and fine PM, as well as those of ultrafine PM, to gain more profound insights into the underlying mechanisms regarding their formation, evolution, and removal processes, which are significant for assessing aerosol climate and health impacts and developing more effective air pollution control strategies.

## Data Availability

Data used in this study are available by request from the corresponding author.

## References

[1] Liang L., Gong P. (2020). Urban and Air Pollution: A Multi-City Study of Long-Term Effects of Urban Landscape Patterns on Air Quality Trends. Sci. Rep..

[2] Fu H., Chen J. (2017). Formation, Features and Controlling Strategies of Severe Haze-Fog Pollutions in China. Sci. Total Environ..

[3] Lu F., Xu D., Cheng Y., Dong S., Guo C., Jiang X., Zheng X. (2015). Systematic Review and Meta-Analysis of the Adverse Health Effects of Ambient PM2.5 and PM10 Pollution in the Chinese Population. Environ. Res..

[4] Wang X., Zhang R., Yu W. (2019). The Effects of PM2.5 Concentrations and Relative Humidity on Atmospheric Visibility in Beijing. J. Geophys. Res. Atmos..

[5] Huang R.J., Zhang Y., Bozzetti C., Ho K.F., Cao J.J., Han Y., Daellenbach K.R., Slowik J.G., Platt S.M., Canonaco F. (2014). High Secondary Aerosol Contribution to Particulate Pollution during Haze Events in China. Nature.

[6] An Z., Huang R.J., Zhang R., Tie X., Li G., Cao J., Zhou W., Shi Z., Han Y., Gu Z. (2019). Severe Haze in Northern China: A Synergy of Anthropogenic Emissions and Atmospheric Processes. Proc. Natl. Acad. Sci. USA.

[7] Li Y., Xue Y., Guang J., de Leeuw G., Self R., She L., Fan C., Xie Y., Chen G. (2019). Spatial and Temporal Distribution Characteristics of Haze Days and Associated Factors in China from 1973 to 2017. Atmos. Environ..

[8] Seinfeld J.H., Pandis S.N. (2006). Atmospheric Chemistry and Physics: From Air Pollution to Climate Change.

[9] Bei N., Li G., Huang R.J., Cao J., Meng N., Feng T., Liu S., Zhang T., Zhang Q., Molina L.T. (2016). Typical Synoptic Situations and Their Impacts on the Wintertime Air Pollution in the Guanzhong Basin, China. Atmos. Chem. Phys..

[10] Wang Y.C., Huang R.J., Ni H.Y., Chen Y., Wang Q.Y., Li G.H., Tie X.X., Shen Z.X., Huang Y., Liu S.X. (2017). Chemical Composition, Sources and Secondary Processes of Aerosols in Baoji City of Northwest China. Atmos. Environ..

[11] Zhou B., Wang Q., Zhou Q., Zhang Z., Wang G., Fang N., Li M., Cao J. (2018). Seasonal Characteristics of Black Carbon Aerosol and Its Potential Source Regions in Baoji, China. Aerosol Air Qual. Res..

[12] Xiao S., Wang Q.Y., Cao J.J., Huang R.J., Chen W.D., Han Y.M., Xu H.M., Liu S.X., Zhou Y.Q., Wang P. (2014). Long-Term Trends in Visibility and Impacts of Aerosol Composition on Visibility Impairment in Baoji, China. Atmos. Res..

[13] Xie M., Wang G., Hu S., Han Q., Xu Y., Gao Z. (2009). Aliphatic Alkanes and Polycyclic Aromatic Hydrocarbons in Atmospheric PM10 Aerosols from Baoji, China: Implications for Coal Burning. Atmos. Res..

[14] Grimm H., Eatough D.J. (2009). Aerosol Measurement: The Use of Optical Light Scattering for the Determination of Particulate Size Distribution, and Particulate Mass, Including the Semi-Volatile Fraction. J. Air Waste Manag. Assoc..

[15] Draxier R.R., Hess G.D. (1998). An Overview of the HYSPLIT_4 Modelling System for Trajectories, Dispersion and Deposition. Aust. Meteorol. Mag..

[16] Wang Y.Q., Zhang X.Y., Draxler R.R. (2009). TrajStat: GIS-Based Software That Uses Various Trajectory Statistical Analysis Methods to Identify Potential Sources from Long-Term Air Pollution Measurement Data. Environ. Model. Softw..

[17] Guan Q., Yang Y., Luo H., Zhao R., Pan N., Lin J., Yang L. (2019). Transport Pathways of PM10 during the Spring in Northwest China and Its Characteristics of Potential Dust Sources. J. Clean. Prod..

[18] Luo H., Guan Q., Pan N., Wang Q., Li H., Lin J., Tan Z., Shao W. (2020). Using Composite Fingerprints to Quantify the Potential Dust Source Contributions in Northwest China. Sci. Total Environ..

[19] Sun J., Shen Z., Cao J., Zhang L., Wu T., Zhang Q., Yin X., Lei Y., Huang Y., Huang R.J. (2017). Particulate Matters Emitted from Maize Straw Burning for Winter Heating in Rural Areas in Guanzhong Plain, China: Current Emission and Future Reduction. Atmos. Res..

[20] Kerminen V.M., Chen X., Vakkari V., Petäjä T., Kulmala M., Bianchi F. (2018). Atmospheric New Particle Formation and Growth: Review of Field Observations. Environ. Res. Lett..

[21] Peng Y., Liu X., Dai J., Wang Z., Dong Z., Dong Y., Chen C., Li X., Zhao N., Fan C. (2017). Aerosol Size Distribution and New Particle Formation Events in the Suburb of Xi’an, Northwest China. Atmos. Environ..

[22] Wang T., Xue L., Brimblecombe P., Lam Y.F., Li L., Zhang L. (2017). Ozone Pollution in China: A Review of Concentrations, Meteorological Influences, Chemical Precursors, and Effects. Sci. Total Environ..

[23] Liu Z., Wang Y., Gu D., Zhao C., Huey L.G., Stickel R., Liao J., Shao M., Zhu T., Zeng L. (2012). Summertime Photochemistry during CAREBeijing-2007: RO x Budgets and O3 Formation. Atmos. Chem. Phys..

[24] Liu P., Ye C., Xue C., Zhang C., Mu Y., Sun X. (2020). Formation Mechanisms of Atmospheric Nitrate and Sulfate during the Winter Haze Pollution Periods in Beijing: Gas-Phase, Heterogeneous and Aqueous-Phase Chemistry. Atmos. Chem. Phys..

[25] Sun W., Shao M., Granier C., Liu Y., Ye C.S., Zheng J.Y. (2018). Long-Term Trends of Anthropogenic SO_2_, NO_x_, CO, and NMVOCs Emissions in China. Earth’s Future.

[26] Li K., Jacob D.J., Liao H., Zhu J., Shah V., Shen L., Bates K.H., Zhang Q., Zhai S. (2019). A Two-Pollutant Strategy for Improving Ozone and Particulate Air Quality in China. Nat. Geosci..

[27] Li G., Bei N., Tie X., Molina L.T. (2011). Aerosol Effects on the Photochemistry in Mexico City during MCMA-2006/MILAGRO Campaign. Atmos. Chem. Phys..

[28] Cheng Y., He K.B., Du Z.Y., Zheng M., Duan F.K., Ma Y.L. (2015). Humidity Plays an Important Role in the PM2.5 Pollution in Beijing. Environ. Pollut..

[29] Zhai S., Jacob D.J., Wang X., Shen L., Li K., Zhang Y., Gui K., Zhao T., Liao H. (2019). Fine Particulate Matter (PM2.5) Trends in China, 2013–2018. Separating Contributions from Anthropogenic Emissions and Meteorology. Atmos. Chem. Phys..

